# Antioxidant Stress and Anti-Inflammation of PPAR**α** on Warm Hepatic Ischemia-Reperfusion Injury

**DOI:** 10.1155/2012/738785

**Published:** 2012-11-11

**Authors:** Zhixin Gao, Yuan-Hai Li

**Affiliations:** Department of Anesthesiology, First Affiliated Hospital of Anhui Medical University, Hefei 230022, China

## Abstract

Hepatic ischemia-reperfusion (IR) injury is a serious clinical problem. Minimizing the adverse effect of ischemia-reperfusion injury after liver surgery or trauma is an urgent need. It has been proved that besides the effect of regulating the lipid and lipoprotein metabolism, PPAR**α** also undertakes the task of organ protection. In this paper, related literature has been summarized and we come to the conclusion that administration of PPAR**α** agonists can strengthen the antioxidant and anti-inflammation defense system by the upregulation of the expression of antioxidant enzymes and inhibition of NF-**κ**B activity. This may provide a potential clinical treatment for hepatic ischemia-reperfusion injury.

## 1. Introduction

Hepatic ischemia reperfusion (IR) injury is an important clinical problem complicating liver surgery and transplantation [1]. Depending on whether the ischemia occurs in a warm setting, such as surgical resection or trauma surgery, or cold ischemia, as occurs during liver transplantation, it can be categorized into warm IR and cold storage reperfusion injury [2]. In warm IR, the pathophysiology underlying the injury of hepatic ischemia-reperfusion is complex encompassing a number of mechanisms including oxidant stress, inflammation, and apoptosis. Recently, some researchers have focused on the use of peroxisome proliferator-activated receptor alpha (PPAR*α*) agonist to ameliorate this injury [3, 4]. In this paper, the mechanisms of hepatic ischemia-reperfusion injury, the characteristics of PPAR*α*, and the role of PPAR*α* in warm hepatic ischemia-reperfusion injury have been discussed in the following sections. 

## 2. Mechanisms of Warm Hepatic Ischemia-Reperfusion Injury

With the growth in the field of hepatobiliary surgery, the technique of partial or total vascular occlusion in room temperature has been adapted, and it has enabled surgeons to perform complex procedures such as large liver resections and repairs that otherwise would have resulted in massive hemorrhage and certain death. Apart from the apparent superiority of the technique, there are still some limitations that can cause substantial morbidity and mortality named warm hepatic ischemia-reperfusion injury. Warm hepatic ischemia-reperfusion injury is a complex cascade of events involving a multitude of pathophysiological processes, more than 50% of hepatocytes and sinusoidal endothelial cells (SEC) that formerly considered to undergo apoptosis during the first 24 hours of reperfusion [5, 6]; however, work done by team of Jaspreets Gujral suggested that apoptotic cell death, if it occurs at all, is a very minor aspect of the entire cell death [7, 8]. Based on it we can conclude that the oxidant stress and inflammation are the most critical mechanisms which contribute to the organ pathophysiology after warm hepatic ischemia reperfusion. Work done by Jaeschke et al. [9–12] indicated that there are two distinct phases of liver injury after warm ischemia and reperfusion. The initial phase of injury (<2 hours after reperfusion) is characterized by Kupffer cells activated, and the activated Kupffer cells are a primary source of reactive oxygen-derived free radicals [10, 13]. These free radicals and reactive oxygen species (ROS) are generated to create a severe enough disturbance of the cellular homeostasis. Mitochondria must be a primary target, and its dysfunction may impair the electron flow and enhance superoxide formation [14, 15]. All these will eventually trigger mitochondrial dysfunction and oxidant stress and eventually kill the cell [16, 17]. Studies have shown that it attenuates early hepatocellular injury after hepatic IR that Kupffer cells activity is suppressed by gadolinium chloride or methyl palmitate in mice [18]. Conversely, chemically upregulating Kupffer cell activation aggravates cellular injury and production of reactive oxygen species [19]. In addition, complement is a key factor that contributes to the early activation of Kupffer cells after IR [20]. Kupffer cell generation of superoxide has been shown to be a decisive factor in the injury observed in the early reperfusion period [20, 21]. In addition to Kupffer cell-induced oxidant stress, with increasing length of the ischemic episode, intracellular generation of reactive oxygen by xanthine oxidase and, in particular, mitochondria [22] may also lead to impaired adenosine triphosphate (ATP) production and acidosis result in liver dysfunction and cell injury during reperfusion [23]. Nevertheless, liver architecture assessed histologically shows only minor changes during the period of ischemia and early reperfusion. In the late phase of injury (>6 hours after reperfusion), events occurring during the initial phased serve to initiate and propagate a complex inflammatory response that culminates with the hepatic accumulation of neutrophils [24]. Kupffer cells which can not only directly activate and recruit neutrophils but also serve as the principal source of the oxidant stress during the first period phase of reperfusion injury, the production, and the release of reactive oxygen species can lead to an oxidative shift in the hepatic redox state [10, 11, 25], that is thought to activate redox-sensitive transcription factor NF-*κ*B, which provides the signal for activation of proinflammatory genes, such as IL-12 and TNF-*α* [26–29]. Productions of these mediators lead to inducing the expression of secondary mediators, including neutrophil-attracting CXC chemokines and endothelial cell adhesion molecules which mediate the adhesion and transmigration of neutrophils from the vascular space into the hepatic parenchyma [30–32]. Neutralizing antibodies to CXC chemokines proven to be effective against neutrophil-induced liver injury during ischemia reperfusion [33] and partial hepatectomy [34]. The priming of neutrophils during this time may be an important factor for the later neutrophil-induced injury phase [11]. Activated neutrophils generate two major cytotoxic mediators, that is, reactive oxygen species and proteases [21]. In addition to the NADPH oxidase-derived superoxide and its dismutation product hydrogen peroxide, data from Tadashi Hasegawa and his co-workers provide a direct evidence for a specific neutrophil-mediated oxidant stress [hypo-chlorite (HOCl)-modified epitopes] during reperfusion when a relevant number of neutrophils have extravasated into the parenchyma from sinusoids [21]. HOCl, generated only from H_2_O_2_ and Cl^−^ by myeloperoxidase (MPO), can diffuse into hepatocytes and cause formation of chloramines, which are potent oxidants and cytotoxic agents involved in hepatocytes killing and responsible for maintaining the inflammatory response [35]. In addition, neutrophils store various proteases in granules and can release these proteolytic enzymes during activation. Protease inhibitors are shown to attenuate neutrophil-induced liver injury [36]. Moreover, reactive oxygen species are indispensable for a protease-mediated injury mechanism under in vivo conditions. Therefore, accumulated neutrophils release oxidants and proteases that directly injure hepatocytes and vascular endothelial cells and may also obstruct hepatic sinusoids resulting in hepatic hypoperfusion [37]. During the second phase of reperfusion injury, neutrophils work as the most acute cytotoxic inflammatory cells activated and recruited, and the damage caused by neutrophils is recognized as a major mechanism of during reperfusion [24, 38, 39]. A recent study by Beraza et al. has suggested that the hepatic inflammatory response to IR is driven largely by NF-*κ*B activation in hepatocytes [40]. Nuclear factor (NF)-*κ*B is a broad term used to describe a number of dimeric combinations of proteins of the Rel family [41, 42]. In unstimulated cells, NF-*κ*B is sequestered in the cytoplasm by inhibitors of *κ*B (I*κ*B) proteins which prevent nuclear localization of NF-*κ*B by masking its nuclear localization signal peptide and block NF-*κ*B from binding to DNA by allosteric inhibition [43]. Once is freed from I*κ*B, NF-*κ*B translocates to the nucleus where it initiates the transcription of target genes such as tumor necrosis factor-a (TNF-a), interleukin (IL)-1b, and IL-6 [24, 44, 45]. The NF-*κ*B inhibitory protein A20 demonstrates hepatoprotective abilities through curtailing inflammation by inhibiting NF-*κ*B activation [46]. On the contrary, A20 knockout mice are born cachectic and die within 3 weeks of birth as a result of unfettered liver inflammation [47]. Therefore, above the literature provides compelling evidence that inhibition of oxidant-stress and inflammation in hepatocytes during IR injury is an essential mechanism of protection.

## 3. Characteristics of PPAR**α**

PPAR is originally identified by Issemann and Green [48] after screening the liver cDNA library with a cDNA sequence located in the highly conserved C domain of nuclear hormone receptors. A notability subtype of PPAR will be discussed here is PPAR*α*. In human body, PPAR*α* gene which spans ~93.2 *κ*b is located on chromosome 22q12-q13.1 and encodes a protein of 468 amino acids [49]. While in mice, PPAR*α* gene is located on chromosome 15E2, and it also encodes a protein of 468 amino acids [50]. PPAR*α* contains four major functional domains, which are the N-terminal ligand-independent transactivation domain (A/B domain), the DNA binding domain (DBD or C domain), the cofactor docking domain (D domain), and the C-terminal E/F domain (including the ligand binding domain (LBD) and the ligand-dependent transactivation domain (AF-2 domain) [51, 52]. The divergent amino acid sequence in the LBD of PPAR*α* is thought to provide the molecular basis for ligand selectivity. A large ligand-binding pocket (1300 Å) exists in PPAR*α*, allowing diverse and structurally distinct compounds access to the LBD [51] and enabling PPAR*α* to sense a broad range of endogenous substances, including fatty acids and their derivatives, or exogenous ligands, such as fibrates, Wy14643 [53, 54], and so on. Analogous with several other nuclear hormone receptors, PPAR*α* also is a ligand-activated transcription factor which upon heterodimerization with the retinoic X receptor (RXR), recognizes PPAR response elements (PPRE), located in the promoter of target genes [55]. PPAR*α* is highly expressed in the liver, kidney, and heart muscle, which are all organs that possess high mitochondrial and *β*-oxidation activity. PPAR*α* basically function, as sensor for fatty acid derivatives and controls essential metabolic pathways involved in lipid and energy metabolism, and it also plays a significant role in various pathophysiologic conditions, such as inflammation and apoptosis caused by injury [56]. Our group and others have demonstrated that PPAR*α* is an important regulator of postischemic liver injury [3, 4].

## 4. Roles of PPAR**α** on Warm Hepatic Ischemia-Reperfusion Injury

**Figure 1:**
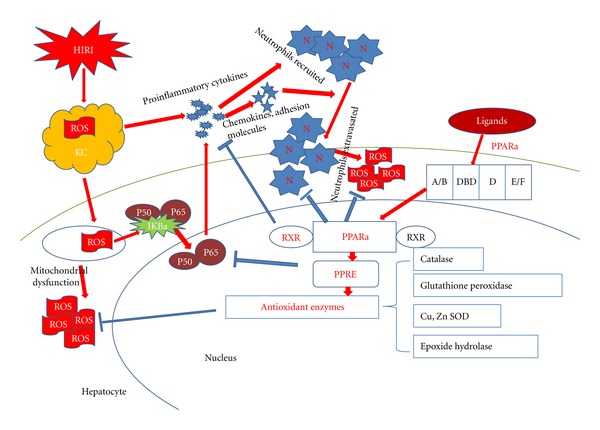
Protection mechanism of PPAR*α* in the liver during IR injury. Ischemic stress results in the generation of reactive oxygen species (ROS) in Kupffer cells. ROS activates NF-*κ*B and induces mitochondrial dysfunction in neighboring hepatocytes. Activation of NF-*κ*B consequences in the production of proinflammatory cytokines, chemokines and adhesion molecules which can recruit neutrophils and propagate the inflammatory response. This vicious circle is breaked by PPAR*α* which is a ligand-activated transcription factor that upon heterodimerization with the retinoic X receptor (RXR), recognizes PPAR response elements (PPRE), located in the promoter of target genes. Abbreviations: Neutrophil (N), Kupffer cell (KC).

Reactive oxygen species and inflammation factors are critical mediators that exert a toxic effect during warm hepatic ischemia-reperfusion injury. Therefore, the most significant mechanisms of PPAR*α* hepatoprotective abilities have been demonstrated through antioxidant stress and anti-inflammation functions (Figure 1). 

### 4.1. Antioxidant Stress

Interruption of blood flow to liver and subsequent reperfusion lead to an acute oxidant stress response that may cause significant cellular damage and organ dysfunction. Hepatocellular injury during both the initial and later phases of reperfusion is caused in large part by reactive oxygen species including hypochlorite (HOCl) [10, 11, 25]. Accordingly, antioxidant therapy can limit the ischemia-reperfusion injury [57, 58]. The discovery of the colocalization of catalase with H_2_O_2_-generating oxidases in peroxisomes is the chief indication of their involvement in the metabolism of oxygen metabolites [59]. Peroxisomes, which are subcellular organelles within the hepatocyte, contain a battery of antioxidant enzymes and may help protect hepatocytes from oxidative damage. Catalase is the classical marker enzyme of peroxisomes metabolizing both H_2_O_2_ and a variety of substrates such as ethanol, methanol, phenol, and nitrites by peroxidatic activity [60], so it plays an important protective function against the toxic effects of peroxides and removes them with high efficiency [61]. PPAR*α* stimulation by Wy14643 induces expression and activation of antioxidant enzymes such as superoxide dismutase (SOD), catalase, and glutathione (GSH), which protects hepatocytes against hepatic IR injury mice model in vivo [3]. From the vitro experimental results of our group, the protective effect of Wy14643 is demonstrated herein by reducing ALT, AST, ROS levels and ameliorating ultrastructure alterations of hepatocytes; this protection is associated with an inhibition of oxidative stress and upregulation of hepatocytes PPAR*α*-mRNA expression [62]. PPAR*α* has also been implicated in the expression or activation of antioxidant enzymes such as catalase and Cu_2_^+^, Zn_2_^+^ superoxide dismutase (SOD1) [63]. Work done by Tetsuya Toyama has indicated that PPARa ligands (WY14643) play an antifibrotic action through disrupting the vicious cycle between hepatic damage and oxidative stress by activating antioxidant enzymes such as catalase, and resolving the oxidative stress in the rat TAA model of liver cirrhosis [63]. The PPAR*α* agonist is also highly effective in the treatment of dietary steatohepatitis in mice which results from the action of ROS on accumulated lipids and excessive formation of lipoperoxides in the liver [64]. Simultaneously, the improved antioxidant defense system after PPAR*α* activation can thus additionally protect against neutrophil cytotoxicity, and the related mechanism can be concluded that formation of ROS plays a key role in leading to hepatocytes necrosis mediated by neutrophil. After mice with 90 min ischemia and 8 h reperfusion, Tomohisa Okaya and Alex B. Lentsch have suggested that PPAR*α* ^−/−^ mice have augmented hepatocellular injury compared with wild-type mice, which is proved to be associated with a marked increase in the amount of neutrophils recruited to the liver, because at this time point much of the injury to hepatocytes is thought to be due to reactive oxygen species and proteases released from recruited neutrophils. On the contrary, treatment of C57BL/6 mice with 10 mg/kg iv WY-14643 1 h before ischemia resulted in a modest but significant reduction in hepatocellular injury [3]. Furthermore, antioxidants and other interventions directed toward detoxification of reactive oxygen species also attenuated inflammatory liver injury [12, 65–67]. The mechanism of PPAR*α* response to ROS is much more complex that it requirs further research. At the same time, the regulation of antioxidant enzymes is closely related to apoptosis signaling [68]. Therefore, elevation of anti-oxidative enzymes can suppress apoptosis and this can also promote carcinogenesis [69].

### 4.2. Anti-Inflammation

Neutrophils are recruited from the vascular space through a complex series of events that involve upregulated expression of cellular adhesion molecules on hepatic vascular endothelial cells and increased production of CXC chemokines [31, 70]. Accumulated neutrophils release oxidants and proteases that directly and drastically injure hepatocytes and vascular endothelial cells [24]. The first evidence indicating a potential role for PPAR*α* in the inflammatory response is the demonstration that leukotriene B4 (LTB4), a proinflammatory eicosanoid, binds to PPAR*α* and induces the transcription of genes involved in *ω*- and *β*-oxidation which leads to the induction of its own catabolism [71]. In this respect, the activation of PPAR*α* by leukotriene B4 serves to limit the inflammatory process, providing a physiological mechanism to stop the damaging effects associated with inflammation [72]. Numerous recent studies have been aimed at delineating the cellular and molecular mechanisms explaining the control of the inflammatory response by PPAR*α* in hepatic ischemia-reperfusion injury. Primarily according to previous studies by our group, the protective effects of PPAR*α* agonists (WY14643) in postischemic liver injury are possibly associated with reductions in neutrophil accumulation, oxidative stress, and tumor necrosis factor (TNF) and interleukin-1 (IL-1) expression in livers during IR [4]. The results also have been supported by additional experimental results [38, 63]. Furthermore, works done by Tomohisa Okaya and Alex B. Lentsch suggest that livers from PPAR*α* ^−/−^ mice have significantly more postischemic injury compared with those from wild-type mice. A possible reason may be the augmented liver neutrophil accumulation and the modest increases in activation of the transcription factor NF-*κ*B. Treatment of cultured murine hepatocytes with WY-14643, a specific agonist of PPAR*α*, protected cells against oxidant-induced injury. However, there are no differences in proinflammatory mediator production between PPAR*α* ^−/−^ and wild-type mice. These data suggest that PPAR*α* is an important regulator of the hepatic inflammatory response to ischemia reperfusion in a manner that is independent of proinflammatory cytokines [3]. What's more, evidence from in vitro experiments for an anti-inflammatory action of PPAR*α* in endothelial cells and monocytes also demonstrate, that PPAR*α* ligands inhibit cytokine-induced genes, such as expression of vascular cell adhesion molecule-1 and tissue factor by downregulating the transcription of these genes [73–75]. Researches address the molecular mechanisms of this anti-inflammatory action demonstrate that PPAR*α* negatively regulate the transcription of inflammatory response genes by antagonizing the nuclear factor-*κ*B (NF-*κ*B) signaling pathway [76]. In addition to the antagonistic action on NF-*κ*B signalling, PPAR*α* activators are known to induce inhibitor of NF-*κ*B, I*κ*B-*α*, in primary smooth muscle cells and hepatocytes, which is associated with reduced NF-*κ*B DNA binding triggered by PPARa [58]. N-3 polyunsaturated fatty acids (n-3 PUFA), eicosapentaenoic acid (EPA) and docosahexaenoic acid (DHA) are considered as PPAR*α* agonists and can also decrease the expression of proinflammatory genes by preventing I*κ*B phosphorylation and NF-*κ*B translocation into the nucleus [77]. Recent studies have suggested that liver preconditioning against IR injury by n-3PUFA supplementation is mediated by PPAR*α* diminishing NF-*κ*B DNA binding through direct protein-protein interaction with NF-*κ*B subunit p65, leading to the recovery of NF-*κ*B signalling activity and reestablishment of inflammatory cytokine homeostasis [78]. The zinc finger protein A20 [79], which is an intracellular ubiquitin-editing enzyme, plays a significant role in the negative feedback regulation of NF-*κ*B activation in response to a diverse range of stimuli [80]. In lethal liver ischemia-reperfusion injury model, the survival rate of mice treated with A20 reached 67% compared with 10%–25% of control mice injected with saline. This major survival advantage in A20-treated mice is associated with protecting against liver IR injury by increasing hepatic expression of PPAR*α*. A20-mediated protection of hepatocytes from hypoxia/reoxygenation and H_2_O_2_-mediated necrosis is reverted by pretreatment with the PPAR*α* inhibitor MK886 [81]. However, the reverse evidence published by Yu et al. [82] reported that inhibition of NF-*κ*B activation by A20 aggravated partial hepatic ischemia-reperfusion injury. According to this report, Xu et al. suggested that NF-*κ*B inactivation in hepatocytes switches the TNF-*α* response from proliferation to apoptosis, so decreased NF-*κ*B activity sensitizes hepatocytes to TNF-*α*-induced cytotoxicity and may contribute to increased liver dysfunction [83]. In nonalcoholic steatohepatitis (NASH) and simple steatosis, besides reducing steatosis by regulation lipid and lipoprotein metabolism, treatment of mice with the PPAR*α* activator Wy14643 protects steatotic livers against IR injury, with the benefits of this treatment potentially occuring through dampening vascular cellular adhesion molecule-1 and cytokine responses and activation of NF-*κ*B and IL-6 production [84]. The former contradictory results have suggested that the role of NF-*κ*B activation during hepatic ischemia-reperfusion injury is controversial because it induces both protective and proinflammatory genes [85] and NF-*κ*B inactivation either protects against hepatic IR injury [86–88] or aggravates such injury [45, 89, 90]. Studies done by Nozomu Sakai and Heather have indicated that activation of NF-*κ*B in Kupffer cells promotes inflammation through cytokine expression, whereas activation in hepatocytes may be cell protective, based on the fact that they further proved that exogenous administration of receptor activator of NF-*κ*B ligand (RANKL) reduces liver injury in a manner associated with increased hepatocyte NF-*κ*B activation [91]. Supplementary works needed to be completed to explore how the PPAR*α* plays a role in directing a clinical outcome which may lead to better prospects of more rational approaches to reduce postischemic liver injury. In addition to all of the mentioned above, PPAR*α* is also a target of the hypothalamic hormone signaling as it plays an important role in the anti-inflammatory action of glucocorticoids [92].

## 5. Perspectives

As outlined in this paper, there is ample evidence for a critical involvement of oxidant-stress and inflammatory response in various animal models of hepatic ischemia-reperfusion injury. ROS are generated during initial reperfusion, where the initial cell damage triggers an inflammatory response with activation of tissue macrophages and recruitment of neutrophils both of them cause cell death and liver injury. Growing evidences for a role of PPAR*α* in a variety of physiological and pathological processes, particularly the participation in the pathophysiology of inflammation and the protective role of hepatic ischemia-reperfusion injury via limiting oxidative injury as well as inhibiting inflammation response, has emerged in prevenient researches. However, species-specific differences in response to PPAR*α* activators still exist between human and animals. Rats and mice are highly susceptible to peroxisome proliferation and are susceptible to hepatocarcinogenesis due to the antiapoptosis of PPAR*α* activators. Whereas, PPAR*α* agonists are clinically and functionally relevant as fibrate therapeutics against hyperlipidemia and agents for reducing the complications of peripheral vascular disease in diabetic patients [93]. Yet, there are no sufficient evidence to show that hepatic cancer, hypertrophy, or peroxisome proliferation is relevant to it. On the contrary, PPAR*α* receptor activation can interrupt the development process of chronic hepatitis C and NASH to liver cancer by regulating the lipid and lipoprotein metabolism and enhancing the antioxidant stress. This is because obesity-related metabolic abnormalities [94], especially insulin resistance, may be a decisive factor in the pathogenesis of chronic hepatitis C as recently suggested in nonalcoholic steatohepatitis (NASH), along with impairment in lipid metabolism [95]. And the second reason is that oxidative stress not only damages hepatocytes and increases the rate of hepatocyte death, but also inhibits the replication of mature hepatocytes [96]. In order to balance the decreased replication capacity of mature hepatocytes, hepatic progenitors accumulate in the liver; this abnormal regenerative process may contribute to Hepatocellular Carcinoma.

One crucial difference is that the level of expression of PPAR*α* in the human liver is lower than that found in rats and mice [97]. It is also worth pointing out that long term exposure to these drugs can result in oxidative DNA damage, among other effects [92, 98]. So despite their potentially beneficial roles, PPAR*α* agonists should be used judiciously.
